# Inflammable Celluloid

**Published:** 1902-03-01

**Authors:** 


### INFLAMMABLE CELLULOID.

Professor Ogston[1] draws attention to the serious nature of the injuries which are not infrequently caused by burning celluloid, and also to the extreme inflammability of some specimens of this material, especially when it is not properly made. The matter is rendered all the more important in consequence of the growing frequency with which celluloid is employed in the manufacture of combs—particularly imitation tortoiseshell combs—buttons and other ornaments, and the celluloid cuffs and collars which are now so largely used. All these things are full of danger, being liable to take fire on exposure to a very moderate degree of heat. A woman kneeling in front of, but by no means very near to, a clear red fire, and having an imitation tortoiseshell comb in her hair, on the top of her head, became conscious of a smell of burning and a very disagreeable vapour around her, and on putting up her hand found that her head was on fire. She poured water over it and got it out, but not before a great deal of her hair was burnt. In another case the patient was seated in a drawing room before a fire which, though burning well, was not unusually strong, when she found her head enveloped in smoke and flame from the ignition of a comb amongst her hair, and before the flames could be extinguished; she had a portion of her scalp destroyed over an area of perhaps four by one and a-half inches. The burn took many months to heal, and resulted in the total destruction of the hair over the injured area. Professor Ogston, with the assistance of Professor Japp, has made a series of experiments with the object of ascertaining the temperature at which celluloid will take fire when exposed to heat, and he finds that, although in the case of good, well-made celluloid, the temperature at which it will inflame is stated to be 383° Fahr., a far lower temperature will induce this effect with some of the common specimens which are employed in the manufacture of the articles mentioned above. For example, a portion of the comb which caused the accident in one of the cases was placed in contact with the steel of a hat pin, the steel likewise touching the thermometer, and the whole being placed in front of a fire. The moment the thermometer registered 200° Fahr. the celluloid ignited. Another portion of the same comb was enveloped, with a thermometer, in a small lock of the black hair of the patient, and exposed to the radiant heat of the fire. The celluloid in this case took fire when the thermometer marked 180° Fahr. In another case, in which a child's fair hair was used, the celluloid burnt at 167° Fahr. Such cases are sufficient to show how extremely dangerous a head decoration is this inflammable material, and how very important it is that some measures should be taken to prevent the use for the purpose of badly manufactured celluloid, which evidently is liable to catch fire at temperatures far below that which is normal in the case of the properly prepared article. Even with properly manufactured celluloid, although, no doubt, the danger is less, women would be well advised not to decorate their heads with articles made of such an inflammable substance, while certainly for boys and men, who smoke and use matches, to surround their necks and wrists with false cuffs and collars which are liable to go off like fireworks if touched by a spark, is the height of folly.

[1] Lancet, Feb. 22.

